# A Safe and Accessible Cell-Based Spike–ACE2 Binding Assay for Evaluating SARS-CoV-2 Neutralization Activity in Biological Samples Using Flow Cytometry

**DOI:** 10.3390/mps8050104

**Published:** 2025-09-08

**Authors:** Martin A. Rossotti, Shannon Ryan, Greg Hussack, Jamshid Tanha, Bassel Akache, Tyler M. Renner

**Affiliations:** 1 Human Health Therapeutics Research Centre, National Research Council Canada, Ottawa, ON K1A 0R6, Canada; martin.rossotti@nrc-cnrc.gc.ca (M.A.R.); shannon.ryan@nrc-cnrc.gc.ca (S.R.); greg.hussack@nrc-cnrc.gc.ca (G.H.); jamshid.tanha@nrc-cnrc.gc.ca (J.T.); bassel.akache@nrc-cnrc.gc.ca (B.A.); 2 Department of Biochemistry, Microbiology and Immunology, University of Ottawa, Ottawa, ON K1H 8M5, Canada; 3 Center for Infection, Immunity, and Inflammation (CI3), University of Ottawa, Ottawa, ON K1H 8M5, Canada

**Keywords:** flow cytometry, SARS-CoV-2, ACE2, spike, antibodies, nanobodies, serum, neutralization, neutralizing antibodies (nAbs), vaccination

## Abstract

SARS-CoV-2, the agent responsible for coronavirus disease in 2019 (COVID-19), has caused extensive global health and socioeconomic impact due to its transmissibility and pathology. As a result, it was classified as a Risk Group 3 human pathogen, and handling samples containing live virus requires enhanced biological containment facilities (i.e., CL3) to reduce the potential of laboratory infection to personnel and the spread of the virus into the community. While the use of an authentic live virus remains the gold standard for biological assays, alternative methods have been developed to effectively evaluate neutralization activity in the absence of a replicating viral agent. Here, we describe a cell-based spike–ACE2 binding assay as a surrogate for neutralization of SARS-CoV-2 spike to identify potential neutralizing antibodies. A main advantage of this approach is the exclusion of infectious viral particles, increasing biosafety for laboratory personnel. The interaction of recombinant SARS-CoV-2 trimeric spike protein with ACE2 is monitored and quantified by flow cytometry. Notably, our previous studies have demonstrated the utility of this assay for other viruses, beyond SARS-CoV-2. The methodology presented here has exhibited a strong correlation to other widely accepted methods, such as pseudotyped lentiviral and live virus neutralization assays, in identifying neutralizing antibodies.

## 1. Introduction

The gold standard methodology to evaluate neutralization of SARS-CoV-2 involves the use of live coronavirus within the plaque reduction neutralization test (PRNT). Despite its utility, the need for containment level 3 (CL3) and uniquely trained personnel, has limited its use within the research community [1,2,3]. Assays relying on pseudotyped viral vectors displaying the spike protein have emerged as an efficient alternative requiring lower containment level 2 (CL2) infrastructure, given the replication-incompetent design of the vector, allowing only a single-round of infection. Although this technique is very versatile, it is time consuming, requiring generation of the virus, productive infection and downstream expression and readout of a reporter gene [4,5,6]. In both cases, working with the virus (live coronavirus or pseudotyped lentivirus) poses biosafety risks, and it is challenging to standardize between laboratories, given variations in factors such as the number of spike proteins on the viral surface [7,8,9].

Several protein-based surrogate neutralization assays have been developed as alternatives to circumvent the challenges and risks associated with handling infectious coronavirus by employing enzyme-linked immunosorbent assays (ELISAs) [1,7,10,11,12,13], surface plasmon resonance (SPR) [10,12], microfluidic assays [4] or cell-free (bead-based) multiplexed flow cytometry [14,15,16,17]. These assays mimic an essential step in the viral life cycle, whereby SARS-CoV-2 spike (S) or the receptor binding domain (RBD) binds to the host cell using the surface receptor angiotensin-converting enzyme 2 (ACE2). Each surrogate assay offers distinct strengths; for example, cell-free (bead-based) multiplexed flow cytometry and ELISA-based methods excel in throughput, SPR provides affinity and competition data, and microfluidic assays enhance portability while reducing reagent use. Their performance has been compared and validated with PRNT and pseudotyped viral assays, which, though lower in throughput, incorporate authentic viral entry mechanisms dependent on spike protein function. Overall, surrogate assays show a reasonably strong correlation with these reference methods in predicting the neutralization potency of antibodies (Abs).

In most surrogate neutralization assay formats, however, the spike, RBD or ACE2 is adsorbed or chemically conjugated to a surface, which is structurally quite rigid and frequently associated with improper orientation or denaturation of the target molecule, critical parameters that significantly impact the assay performance. This contrasts with cellular assays, which display receptors in their natural state, offering more fluidity with regard to conformation, localization and interactions with protein-binding partners. Vero E6 cells (derived from African green monkey kidney) have been used since the beginning of the COVID-19 pandemic as a representative target cell due its susceptibility to infection by SARS-CoV and SARS-CoV-2. This cell line is widely accessible and represents an affordable source of endogenously expressed ACE2. In spite of Vero E6 encoding a non-human primate ACE2, the high degree of overall protein homology with the human ACE2 [18], which includes an identical set of 12 key amino acids on human ACE2 involved in the interaction with the spike, has enabled its utility in these types of assays [19,20]. These factors make the cell line suitable to predict the blocking capacities of biological samples including antibodies, although other alternative cells are available, such as conventional Vero cells [21], HuH7 [22,23] and genetically modified stable cell lines overexpressing human ACE2 (e.g., HEK-293T-hACE2) [24,25,26,27]. As a result, it is quite commonplace to use Vero E6 or HEK-293T-hACE2 cells within neutralization assays involving infectious SARS-CoV-2 or pseudotyped virus, respectively.

**Figure 1:**
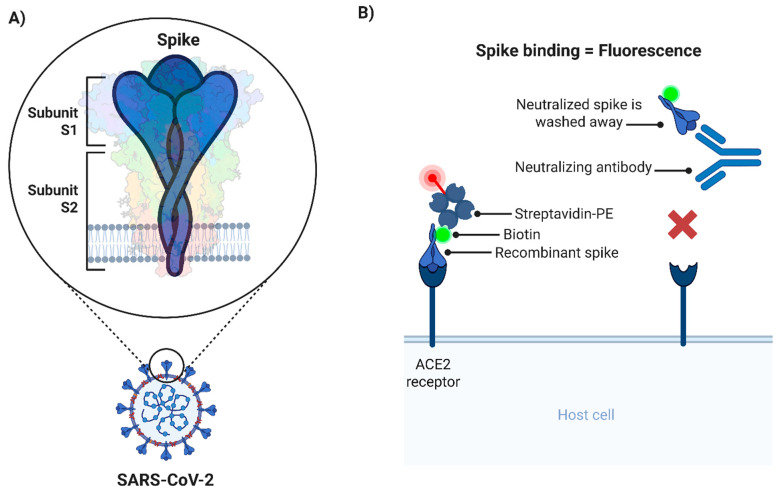
Schematic representation of the cell-based spike–ACE2 binding assay. (**A**) Illustration of the trimeric spike and its subunits expressed in the context of the SARS-CoV-2 viral particle. (**B**) Diagram of the cell-based spike–ACE2 binding assay. Maximum spike binding (and maximum fluorescence) takes place in absence of nAbs (**left**). Conversely, the presence of nAbs will prevent the interaction of biotinylated spike with ACE2, and as a consequence fluorescence is reduced (**right**). This scenario is similar to the background control wells, where no biotinylated spike is added. nAbs: Neutralizing antibodies; PE: R-phycoerythrin.

In this protocol, we describe a flow cytometry-based assay designed to measure the SARS-CoV-2 spike–ACE2 interaction on the cell surface, as an initial screening platform for the classification of antibodies with potential neutralizing activity against SARS-CoV-2. Vero E6 cells act as the source of ACE2, while a chemically biotinylated trimeric spike protein (bio-spike) acts as a surrogate for the virus (Figure 1). A trimeric spike protein was chosen to mimic the structure and conformation of the spike that is displayed on the viral surface. The use of bio-spike, rather than infectious SARS-CoV-2 or a pseudotyped lentivirus, is what enables the assay to be performed under biocontainment level 1 (CL1) conditions. Vero E6 cells are classified as risk group 1 (RG1), further supporting the suitability of this safe and accessible methodology. Thus, the described protocol effectively minimizes biosafety risk to personnel by eliminating the infectious viral component, while maintaining the more natural cell surface microenvironment for the ACE2 receptor.

In our initial optimization studies, similarly to the cell-based ELISA developed by Pi-Estopiñan et al. (2022) [21] and the inhibition flow cytometry-based virus neutralization test developed by Dashti et al. (2024) [28] and Maghsood et al. (2025) [2], we focused on using a fraction of the S protein, the RBD fused to the human IgG_1_ (RBD-Fc) in place of the virus, since most neutralizing antibodies (nAbs) target the RBD. As a result, the assay was limited to the classification of neutralization only by the disruption of the direct RBD–ACE2 interaction. In a second phase, it was hypothesized that Abs targeting regions distal from the RBD may also inhibit the interaction with ACE2. This is achieved by direct steric hindrance of Abs masking the RBD or indirectly by causing allosteric conformational changes on the S protein [29]. In order to provide a substrate that is conformationally similar to the pre-fusion spike on a viral particle, we selected a trimeric, proline-stabilized and furin-cleavage-site-ablated, recombinant spike [30,31]. We found that the use of the full-length trimerized S protein provided a more exhaustive assessment of the neutralization activity and indeed outperformed the RBD as a predictor for neutralization. This new adaptation enabled us to identify nAbs that may have otherwise been missed when assessed by alternative neutralization surrogate assays (ELISA, SPR, and by flow cytometry using RBD-Fc), as neutralization was achieved by targeting the N-terminal domain (NTD) of the spike (i.e., VHHs SR01, SR02) [12]. This is an important consideration especially for discovery of therapeutic nAbs, given the rapid evolutionary escape of the virus by mutations within the RBD. Taken together, the novelty of our approach is based on the cell surface interaction between trimeric spike and ACE2, as it is the most functionally and therapeutically relevant context for the discovery of nAbs.

Using this approach, we profiled a large panel of camelid VHHs targeting the S protein RBD and NTD obtained after the sequential immunization of llamas with full-length S followed by RBD [12]. This platform was also used as a tool to assess the quality of immune responses induced by preclinical SARS-CoV-2 spike vaccines in small animal models [24,25,32,33,34]. To highlight the practical applicability of this methodology, we have easily adapted it for SARS-CoV-2 spikes ranging from ancestral (Wuhan) SARS-CoV-2 to several variants of concern (VOCs), SARS-CoV and several other sarbecoviruses. Furthermore, this assay can be easily benchmarked to positive controls, as we have illustrated previously using the NIBSC 20/136 standard (WHO human standard reference material) [34].

To our knowledge, this is the first flow cytometry-based surrogate neutralization assay that can be performed entirely at CL1 while maintaining trimeric spike–ACE2 interactions in the context of a cellular membrane.

## 2. Experimental Design

### 2.1. Materials

*Commonplace materials are not identified with a supplier or catalogue number.

**In most cases, a functionally equivalent alternative material may be used.

1. 10 mL serological pipettes (Fisher Scientific, Ottawa, ON, Canada; Cat. no.: 356551).
2. 15 mL Falcon tubes (Fisher Scientific, Ottawa, ON, Canada; Cat. no.: 352096).
3. 40 µm mesh strainer (Avantor Mississauga, ON, Canada; Cat. no.: 76327-098).
4. 50 mL Falcon tubes (Fisher Scientific Ottawa, ON, Canada; Cat. no.: 352070).
5. 50 mL plastic reservoir (Sigma Aldrich Oakville, ON, Canada; Cat. no.: AXYRESV50).
6. 96-well U-bottom plates (Thermo Fisher Scientific, Mississauga, ON, Canada; Cat. no.: 168136).
7. 96-well V-bottom plates (Thermo Fisher Scientific, Mississauga, ON, Canada; Cat. no.: 249662).
8. Adhesive plate covers (Diamed, Mississauga, ON, Canada; Cat. no.: DLAU658-2).
9. Amicon Ultra-2 centrifugal filter unit 10 kDa MWCO (MilliporeSigma, Oakville, ON, Canada; Cat. no.: UFC201024).
10. Centrifugal filter unit (MilliporeSigma, Oakville, ON, Canada; Cat. no.: UFC510096).
11. Counting slides (Revvity, Markham, ON, Canada; Cat. no.: CHT4-SD100-002).
12. Glass bottles/beakers (Various N/A).
13. Graduated cylinders (Various N/A).
14. Pipette tips (Various N/A).
15. Serological pipette filler/controller (Various N/A).
16. Spray bottle (Various N/A).
17. SteriTopTM filter unit (0.2 µm) (MilliporeSigma, Oakville, ON, Canada; Cat. no.: SCGPT10RE).
18. Cell culture-treated T75/T175 flasks (Thermo Fisher Scientific, Mississauga, ON, Canada; Cat. no.: 156499/159910).

### 2.2. Equipment

*Commonplace equipment is not identified with a manufacturer or model number.

**In most cases, a functionally equivalent alternative equipment may be used.

1. Analytical balance (Various N/A).
2. Biological safety cabinet (Various N/A).
3. Cellometer Auto 2000 (Revvity, Markham, ON, Canada; Cat. no.: CMT-A2K).
4. Refrigerated centrifuge (Various N/A).
5. Flow cytometer (Various N/A).
6. CO_2_ incubator (Various N/A).
7. Micropipette (Various N/A).
8. Microscope (Various N/A).
9. Milli-Q^®^ IQ 7000 ultrapure water system (MilliporeSigma, Oakville, ON, Canada; Cat. no.: ZIQ7000T0).
10. Multichannel micropipette (Various N/A).
11. NanoDrop™ One/OneC Microvolume UV-Vis spectrophotometer (Thermo Fisher Scientific, Mississauga, ON, Canada; Cat. no.: ND-ONE-W).
12. pH meter (Various N/A).

### 2.3. Reagents

*In most cases, a functionally equivalent alternative reagent may be used. The substitution of the trimeric spike, the biotinylation agent and streptavidin R-phycoerythrin conjugate may impact assay performance which would necessitate optimization.

1. 0.5 M EDTA (Thermo Fisher Scientific, Mississauga, ON, Canada; Cat. no.: 15575020).
2. 10× PBS (Thermo Fisher Scientific, Mississauga, ON, Canada; Cat. no.: 70011044).
3. 2-Mercaptoethanol (Thermo Fisher Scientific, Mississauga, ON, Canada; Cat. no.: 21985023).
4. Accutase^TM^ (Thermo Fisher Scientific, Mississauga, ON, Canada; Cat. no.: A1110501).
5. AOPI (Revvity, Markham, ON, Canada; Cat. no.: CS2-0106-5ML).
6. eBioscience™ IC Fixation Buffer (Thermo Fisher Scientific, Mississauga, ON, Canada; Cat. no.: 00822249).
7. Recombinant trimerized spike protein [31,35] (National Research Council Canada, Montreal, QC, Canada; Cat. no.: SMT1-1).
8. BSA-Fraction V (Rockland Immunochemicals, Limerick, PA, USA; Cat. no.: BSA-1000).
9. Ethanol (Commercial Alcohols, Brampton, ON, Canada; Cat. no.: P016EAAN).
10. EZ-Link™ NHS-LC-LC-Biotin (Thermo Fisher Scientific, Mississauga, ON, Canada; Cat. no.: 21343).
11. FBS (Thermo Fisher Scientific, Mississauga, ON, Canada; Cat. no.: 12483-020).
12. GlutaMAX^TM^ (Thermo Fisher Scientific, Mississauga, ON, Canada; Cat. no.: 35050061).
13. HCl (Fisher Scientific, Ottawa, ON, Canada; Cat. no.: SA48-500).
14. HEPES (Thermo Fisher Scientific, Mississauga, ON, Canada; Cat. no.: 15630080).
15. NaOH (Fisher Scientific, Ottawa, ON, Canada; Cat. no.: SS266-1).
16. Non-essential amino acids (NEAA) (Thermo Fisher Scientific, Mississauga, ON, Canada; Cat. no.: 11140-050).
17. Penicillin-Streptomycin (Thermo Fisher Scientific, Mississauga, ON, Canada; Cat. no.: 15140-122).
18. RPMI 1640 (Thermo Fisher Scientific, Mississauga, ON, Canada; Cat. no.: 21870092).
19. Sodium azide (MilliporeSigma, Oakville, ON, Canada; Cat. no.: S2002-100G).
20. Streptavidin R-phycoerythrin conjugate (SAPE) (Thermo Fisher Scientific, Mississauga, ON, Canada; Cat. no.: SA10044).
21. Trypsin-EDTA (0.05%) (Thermo Fisher Scientific, Mississauga, ON, Canada; Cat. no.: 25300054).
22. Vero E6 cells (ATCC, Manassas, VA, USA; Cat. no.: CRL-1586).

### 2.4. Solution and Standard Preparation

#### 2.4.1. 70% Ethanol

1. Mix 700 mL ethanol with 300 mL Milli-Q water in a plastic spray bottle and store at room temperature indefinitely.

#### 2.4.2. Complete Growth Medium

1. Under aseptic conditions (i.e., in a biosafety cabinet, use serological or micro-pipette) add 50 mL of FBS, 5 mL of HEPES, 5 mL of NEAA, 5 mL of GlutaMAX^TM^, 5 mL of Penicillin–Streptomycin and 500 µL of 2-Mercaptoethanol to a 500 mL bottle of RPMI 1640. Store media at 2–8 °C.

#### 2.4.3. Wash Buffer (PBS + 1% [*w*/*v*] BSA + 0.05% [*w*/*v*] Sodium Azide)


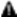
 **CRITICAL STEP:** Inclusion of azide prevents endocytosis of protein when added to cells.

1. Dissolve 10 g of BSA and 0.5 g of sodium azide in 800 mL of Milli-Q water in a glass beaker.
2. Using a graduate cylinder or serological pipette, add 100 mL of 10× PBS and adjust pH to 7.4 with HCl / NaOH as necessary.
3. Top up volume to 1 L with Milli-Q water.
4. Filter sterilize using a 0.2 µm pore size SteriTop^TM^ filter unit. Store solution at 2–8 °C.

#### 2.4.4. Flow Buffer (PBS + 1% BSA + 0.05% Sodium Azide + 5 mM EDTA)

In a new glass bottle, mix 500 mL of the wash buffer prepared in Section 2.4.3. with 5 mL of 0.5 M EDTA. Store solution at 2–8 °C. 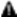 **CRITICAL STEP:** It is important to include EDTA to prevent cellular clumping and potential clogging of the flow cytometer during acquisition; however, do not include EDTA within the wash buffer as it may impact the results.

#### 2.4.5. 1× PBS

Mix 75 mL 10× PBS with 600 mL of Milli-Q water within a 1 L glass bottle.

Adjust pH to 7.4 with HCl/NaOH as necessary and top up volume to 750 mL.

Autoclave under liquid cycle at 121 °C for 30 min to sterilize. Store solution at 2–8 °C.

## 3. Procedure

### 3.1. Thawing and Maintenance of Vero E6 Cells for Assay

3.1.1 Remove a frozen cryovial of Vero E6 cells from liquid nitrogen and thaw in a warm water bath (35–40 °C). Gently shake tube by hand every 15–30 s and ensure tube is removed immediately after ice is thawed or only when a small ice crystal remains. **NOTE**: Avoid contact of the water with the area around the lid of the tube to reduce chances of contamination.
3.1.2 Decontaminate the exterior of the tube with 70% ethanol prior to transferring the tube into a biological safety cabinet (BSC). Use sterile conditions while working within the BSC.
3.1.3 Using a micropipette, transfer the contents of the cryovial into a 15 mL tube containing 10 mL of sterile 1× PBS.
3.1.4 Centrifuge the 15 mL tube at 500× *g* for 5 min at room temperature.
3.1.5 Resuspend the pellet in 10 mL of complete growth medium and transfer the cell-containing medium into a T75 flask with filter cap. 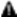 **CRITICAL STEP:** The wash step of 3.1.3 should enable maximal cell growth by removing traces of the freezing media used to cryopreserve the cells.
3.1.6 Maintain Vero E6 cells in a humidified 37 °C incubator with 5% CO_2_. **NOTE:** Vero E6 cells will adhere to the surface and generate a monolayer.
3.1.7 Once 70–90% confluency is reached (≈2–3 days), remove the media using a serological pipette and wash the cells with 10 mL sterile 1× PBS by gentle rotation ensuring the liquid has passed over the entire monolayer. Then, remove the solution by aspiration.
3.1.8 Dispense 1.5 mL of trypsin-EDTA solution into the flask to detach the cells and move back and forth to cover the entire growth area. Incubate for 3–5 min at 37 °C or until cells are dissociated.
3.1.9 Using a serological pipette, add 10.5 mL of complete growth medium to inactivate the trypsin protease solution. Pipette cell suspension up and down 4-6 times to ensure proper dissociation of the cells.
3.1.10 Return a fraction of this solution to the existing or new flask for maintenance of cells. Top up with complete growth medium to 10 mL. **NOTE:** The cells should reach confluence again in 2–3 days if 3 mL of cells from 3.1.9 are added.
3.1.11 Once ready to conduct an experiment, the end user may want to expand Vero E6 culture into multiple T75 flasks (or flasks with larger surface area) depending on number of samples to be analyzed. A confluent T75 flask will provide approximately 10 million cells, which will only be sufficient for a single 96-well plate following the protocol below. Cells may be used for the assay described in 3.3 within 3 months of thawing of cryovial in 3.1.1.

### 3.2. Chemical Biotinylation of Spike

It is important to use a recombinant spike protein with a trimerization domain, such as SMT1-1 used in this study, in order to more closely resemble the native conformation of the viral spike [31,35]. The biotinylation of the recombinant spike is performed following the procedure recommended by the manufacturer using 1 mg of S protein and 20-fold molar excess of NHS-LC-LC-biotin. This format of biotin with a long spacer arm (LC-LC) was chosen to reduce steric hindrance on the spike moiety. Briefly, dissolve 2 mg of biotin in 350 µL DMSO to reach 10 mM solution. Next, add 13.8 µL to 1 mg of spike previously diluted in PBS. Immediately mix by inversion to initiate the reaction. Incubate on ice for 2 h with frequent mixing. No precipitation is expected. The unreacted biotin is removed by several rounds of buffer exchange into PBS using Amicon devices. **OPTIONAL STEP:** Alternative purification methods, such as dialysis, may be used. However, this may impact protein recovery and stability. Quantification of purified product is recommended to account for any loss that may have occurred during buffer exchange. NanoDrop was chosen to quantify purified biotinylated spike protein due to its simplicity and minimal quantity of solution required. Other techniques (i.e., ELISA) may be more suited for enhanced quantification. 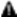 **CRITICAL STEP**: The creation of small single-use aliquots of bio-spike is recommended to avoid freeze–thaw cycles. Long-term storage for up to 2 years at −80 °C is advised. The assay described here was optimized with SMT1-1, substitution of the recombinant trimeric spike and optimal molar ratio for labeling might require additional optimization as it could impact assay performance. The same procedure was used for the biotinylation of the trimeric SARS-CoV and SARS-CoV-2 variants of concern and other sarbecoviruses, obtaining comparable binding activity of each bio-spike to ACE2.

### 3.3. Spike–ACE2 Binding Assay

**Figure 2:**
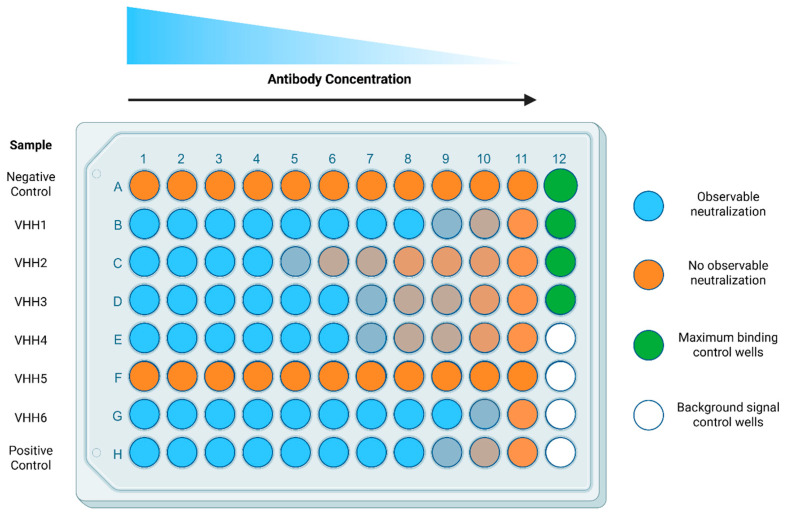
Plate design for the *IC*_50_ determination of nAbs. Biological test samples, potential nAbs or relevant controls, are separated by row (A–H) and titrated by column (1–11). Maximum binding and background signal control wells occupy column 12. All wells receive an equivalent amount of streptavidin conjugated to PE to evaluate the binding of bio-spike. Negative control (Row A) represents an irrelevant VHH (such as VHH A20.1 or A20.1-Fc, specific for *Clostridioides difficile* toxin A) or serum derived from naïve or pre-immunized animals, which should not exhibit specific neutralizing qualities. Positive control (Row H) represents a well-documented neutralizer or soluble ACE2. Maximum binding control wells receive all the reagents except for the biological sample (e.g., potential nAbs or serum). Background signal control wells receive all the reagents except for the biological sample and bio-spike. Shades of orange depict presence of neutralization within the biological samples.

3.3.1 Prepare a 96-well V-bottom plate with the biological samples (e.g., diluted monoclonal antibodies) to be assessed for SARS-CoV-2 spike neutralizing activity. *IC*_50_ (half-maximal inhibitory concentration) determination can be performed by analyzing multiple serial dilutions of the same sample (e.g., serial 3-fold dilutions). Prepare all dilutions in wash buffer. **NOTE:** For each dilution assessed, a final volume of 50 µL remains within each appropriate well of the V-bottom plate.
3.3.2 On each plate, a minimum of 8 wells are set aside for control conditions (Figure 2): four background control wells for measurement of background signal (i.e., cells incubated with wash buffer) and four maximum binding control wells for measurement of maximum spike binding to cells (with bio-spike but no biological test sample). At this stage, simply add 50 µL of wash buffer in these control wells. 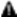 **CRITICAL STEP**: The inclusion of these wells is essential for the calculation of the % of neutralization. **NOTE**: The background control wells do not receive bio-spike in step 3.3.11, while the maximum binding control wells do not receive biological sample (potential nAbs), allowing for minimal and maximal binding respectively.
3.3.3 Remove the flask containing Vero E6 cells from the incubator and place it in the biological cabinet. Work under aseptic conditions to protect the flask from contamination.
3.3.4 As described in step 3.1.7, remove the media and wash the cells with 1× PBS.
3.3.5 Next, remove the PBS and add Accutase^TM^ with a serological pipette to dissociate the cells from the flask (1.5 mL or 4 mL for a T75 or T175 flask, respectively). 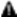 **CRITICAL STEP**: Accutase^TM^ treatment is gentler on the cells than trypsin, helping preserve their health and most importantly to maintain the integrity of membrane-bound proteins such as ACE2, which is fundamental for the surrogate neutralization assay to work.
3.3.6 Incubate cells with Accutase^TM^ at room temperature until cells have largely dissociated (this should take ≈5–10 min). The cell dissociation progress can be monitored under a light microscope.
3.3.7 Add 10.5 mL of complete growth medium per flask and pipette cell suspension up and down 4–6 times to ensure proper dissociation of the cells before transferring into a 15 mL conical tube.
3.3.8 Sub-culture the cells as described in 3.1.10 and return the flask to the incubator if desired.
3.3.9 Centrifuge the cells from step 3.3.7 at 500× *g* for 5 min at room temperature to remove any trace amounts of Accutase^TM^. **OPTIONAL STEP**: For the Vero E6 cells to be used in this assay, aseptic conditions are recommended but not required going forward.
3.3.10 Prepare a 5.5 mL solution of bio-spike at 5 µg/mL in wash buffer per 96-well plate of samples while cells are being centrifuged.
3.3.11 Pour this solution into a 50 mL reservoir and transfer 50 µL of bio-spike into each appropriate well on the 96-well plate already containing your test biological samples using a multichannel micropipette. 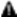 **CRITICAL STEP:** Do not include bio-spike solution in the background control wells (signal measured in the presence of cells and SAPE only) as described in step 3.3.2 and Figure 2. Instead add 50 µL wash buffer.
3.3.12 Use a serological pipette to resuspend the pelleted cells (from step 3.3.9) in complete growth medium (no more than 5 mL for a T75 flask). **OPTIONAL STEP:** Filter the cell solution through a 40 µm mesh strainer into a 50 mL conical tube to remove clumps of cells.
3.3.13 Count the cells by mixing 20 µL of the filtered cell solution with an equal volume of AOPI solution. Mix well and pipette 20 µL of the mixture onto a counting slide to enumerate cells with the Cellometer Auto 2000, as per manufacturer’s instructions.
3.3.14 Adjust cell volume to a final concentration of 2 × 10^6^ viable cells/mL based on the live cell count.
3.3.15 Pour cell solution into a reservoir and transfer 50 µL (1 × 10^5^ cells) into each of the prepared wells of the 96-well plate containing the test samples and controls using a multichannel micropipette. At this point, each well should have a final volume of 150 µL. **NOTE:** Keep in mind that the final dilution of the sample will be 1/3 of the initial prepared dilution in step 3.3.1. Each well will contain 150 µL of solution, 250 ng of bio-spike (except for background controls wells) and 1 × 10^5^ cells.
3.3.16 Cover the prepared plate with an adhesive plate cover and incubate in the fridge (2–8 °C) for 1 h.
3.3.17 Centrifuge the 96-well V-bottom plate for 8 min at 400× *g* at 4 °C.
3.3.18 Remove the adhesive plate cover and remove the supernatant with a multichannel. Gently blot the plate on a paper towel before returning to an upright position. **OPTIONAL STEP:** A quick and confident motion and flicking the plate over a basin is an efficient and practical way to achieve the same result and save the user’s time. **NOTE:** Properly decontaminate the basin and waste fluid to eliminate any cells or biological material at the end of the procedure.
3.3.19 Pour wash buffer into a 50 mL reservoir and add 150 µL to each well with a multichannel micropipette. Dispensing carefully, one can use the same set of tips for all the wells. **OPTIONAL STEP:** Resuspending the cells is not necessary here.
3.3.20 Repeat steps 3.3.17–3.3.19 one more time for a total of 2 washes and then centrifuge to remove the wash buffer as described in 3.3.17. 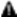 **CRITICAL STEP:** The washes are necessary to remove any trace amount of free bio-spike and or Ab that could interfere in the subsequent step.
3.3.21 Prepare a 1/600 dilution of streptavidin R-phycoerythrin conjugate (SAPE) in wash buffer. For each plate, add 27.5 µL of SAPE (stock at 1 mg/mL) to 16.5 mL of wash buffer in a 50 mL Falcon tube and mix well. **NOTE:** SAPE is used as a probe and will interact with any biotin present on the bio-spike.
3.3.22 Pour the SAPE solution in a reservoir and add 150 µL of solution with a multichannel micropipette to each well. Mix with gentle pipetting using new tips for each well.
3.3.23 Cover the plate with the adhesive plate cover and wrap in aluminum foil. Incubate for 1 h in the fridge (2–8 °C). 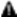 **CRITICAL STEP:** PE is sensitive to light, the aluminum will minimize photobleaching and loss of fluorescence that will impact the sensitivity of the assay.
3.3.24 Repeat steps 3.3.17–3.3.20 to wash away any excess SAPE.
3.3.25 Add 50 µL of Fixation Buffer to each well and mix by gentle pipetting, using a multichannel pipette and new tips for each well. OPTIONAL STEP: We use eBioscience IC Fixation Buffer, but other formaldehyde-based fixatives could also be used.

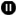
 **PAUSE STEP:** The fixation would allow for up to one week to read the plate with no expected impact on data quality. Alternatively, resuspend cells in 150 µL of flow buffer and read immediately, skipping ahead to 3.3.28.

**Figure 3:**
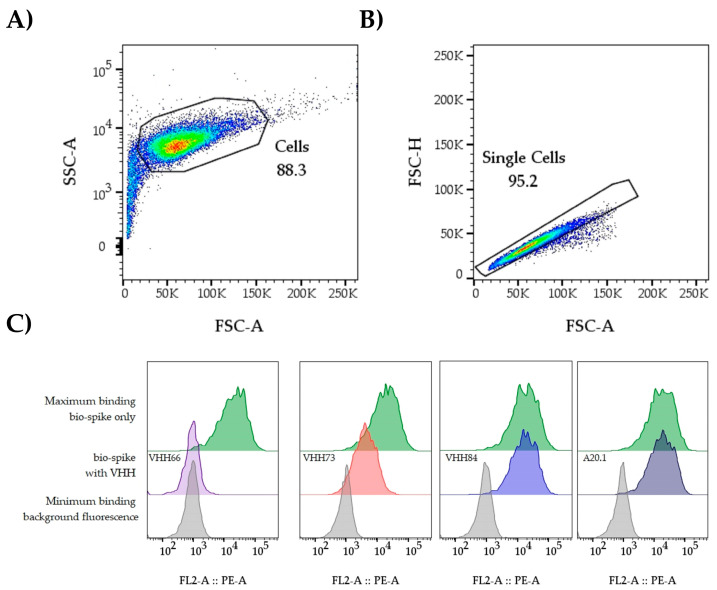
Gating strategy. The data from individual wells is analyzed in FlowJo or a comparable software package. (**A**) The first steps involve the identification and gating of the cell population (FSC-A vs. SSC-A). This is useful to remove elements of smaller/bigger size that could reduce the quality of the data. (**B**) Next, by plotting FSC-A vs. FSC-H we identify the singlet population. The GMFI of PE is extracted from the singlet population. An example of expected data outcomes for the *IC*_50_ analysis workflow is included. Up to six samples can be tested per plate (Figure 2) when including a negative and positive control sample. Starting from 3.3 µg/mL at the highest concentration followed by 3-fold dilutions, it allows the user to cover a range that facilitates obtaining a dose–response curve. (**C**) Representative histograms and outcome. Background and bio-spike controls establish the assay window; potential nAbs can be categorized as non-neutralizing (VHH84), moderate (VHH73), or strong neutralizers (VHH66). A20.1 was included as a negative control.

**Figure 4:**
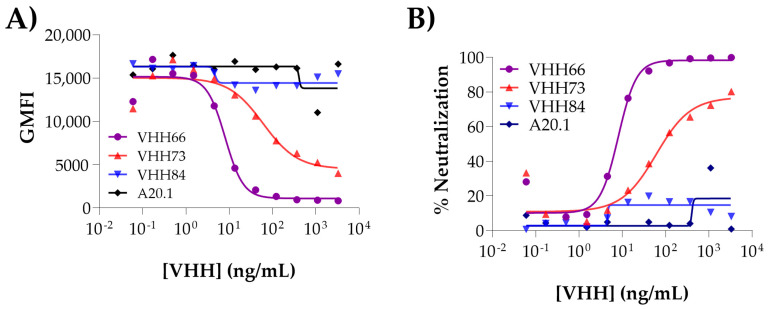
Dose–response titration curves. (**A**) By plotting the raw GMFI values vs. concentration of Ab tested, it is possible to observe at first glance the inhibitory effect of certain Abs. More reliable data (independent of the GMFI, which might vary within assays) is obtained by applying the formula on 3.3.31 to calculate the % of Neutralization value for each Ab tested. The resulting graph is shown in (**B**). The *IC*_50_ is determined using GraphPad (Prism 10 for Windows 64- bit (Version 10.4.1(627), 18 November 2024). Prism by fitting the binding data to non-linear regression [inhibitor] vs. response, variable slope (four parameter) model. The analysis allows the user to classify the Abs as non-inhibitory (VHH84), medium (VHH73) and strong (VHH66) neutralizers. A20.1 is the non-neutralizing VHH included as negative control. The raw data used in the generation of the plots can be found in the Supplementary Materials.

3.3.26 Wrap plates in aluminum foil and incubate in the fridge for 30 min.
3.3.27 Add an additional 100 µL of flow buffer to each well with gentle pipetting. Wrap plates in aluminum foil. **OPTIONAL STEP**: The volume can be reduced to accelerate the acquisition of the data, which usually takes ≈1 h/plate.
3.3.28 **OPTIONAL STEP**: It is recommended to transfer the cells into a 96-well U-bottom plate to reduce chances of clogging the instrument before moving on to acquisition.
3.3.29 Acquire data on a high throughput sampler-capable flow cytometer. Plot the data of relative PE fluorescence on singlets (linear diagonal gating on a dot plot with FSC-H by FSC-A (Figure 3B)). Acquire at most half of the volume (50–75 µL) to allow for a second run if needed (acquiring 10,000 cells should be sufficient for analysis purposes
3.3.30 Using FlowJo™ (BD Life Sciences) or a similar software package, determine the Geometric Mean Fluorescent Intensity (GMFI) of PE for single cells in each well (Figure 4).
3.3.31 Calculate the % Neutralization value using the following formula:

(1) $$\%Neutralization=100-\left(100\times \frac{GMFI\;Sample-GMFI\;Background}{GMFI\;Maximum-GMFI\;Background}\right)$$

where

*GMFI Sample* is the measured fluorescence at any given competitor concentration;
*GMFI Background* is the background fluorescence measured in the presence of cells and SAPE only;
*GMFI Maximum* is the maximum fluorescence measured in the absence of competitor relative to bio-spike-only wells (maximum) and Vero E6-only with SAPE (background).

For background and maximum wells, an average value is used for these replicates.

Samples below or above the expected range (0–100%) can be corrected to the limits of 0% and 100%, respectively. An example of the raw data used in the generation of the plots can be found in the Supplementary Materials.

## 4. Expected Results

This protocol describes the steps to evaluate the activity of biological samples with potential to neutralize SARS-CoV-2 spike glycoprotein trimers from binding to its cognate receptor, ACE2, on the cell surface (Figure 1). As described, the procedure allows for an assessment of SARS-CoV-2 spike neutralization without handling the actual infectious virus allowing the method to be performed entirely at CL1.

In this assay a chemically biotinylated spike, referred to throughout as bio-spike, served as the probe, whereby its interaction with ACE2 is quantified through streptavidin-fluorochrome detection. We selected R-phycoerythrin (PE) owing to its bright fluorescence and therefore greater sensitivity compared to other fluorochromes. Although alternatives to PE, such as FITC are also suitable, they may reduce the stain index and therefore result in a narrower linear dynamic range for this assay.

Several reagents are available to perform the biotinylation of the spike protein, which mainly differ in the length of the spacer. As described in Section 3.2 Chemical biotinylation of spike, we chose NHS-LC-LC-Biotin as the labeling agent because of its long arm, generating a spacer where the biotin is ≈30.5 Å from the spike. This may reduce steric hindrance on the spike trimer and minimize potential perturbation of the interaction between the bio-spike and ACE2. Under these conditions we found that 250 ng of bio-spike per well containing 100,000 cells was optimal for a balance between the amount of protein used per plate and the signal obtained on the assay. To perform one full 96-well plate, 25 µg of bio-spike is necessary.

When applied as a screening tool for purified monoclonal Abs (VHH or VHH-Fc), initially a single point dilution is evaluated for spike neutralization capacity. Samples are diluted to 10 µg/mL prior to mixing with cells and bio-spike (3.3 µg/mL final in the well). Next, Abs that have exhibited neutralization potential undergo a second round of the assay whereby the sample is serially diluted to obtain the *IC*_50_ of each test Ab (Figure 2). In cases where samples may be more limited, such as in serum isolated from small animals, a single concentration may be assessed. However, the recommended dilution values will depend on the type of sample to be analyzed, which requires previous optimization to ensure samples are within the quantifiable range of the assay. This will depend on the precise nature of the experimental setting. For example, our routine assessments of vaccine-induced neutralization responses in mouse serum typically requires a final dilution of between 1/25 and 1/300 (after mixing with cells and bio-spike) [24,25,32,33,34].

As described in 3.3.2, the maximal binding of bio-spike is assumed to take place in the absence of any biological test sample, and the minimal binding occurs without the bio-spike (Figure 1B). These two controls are essential for the calculation of the % of Neutralization value (see formula in 3.3.31) and must be included on every plate to be assayed.

The specificity of the inhibition in the assay is validated by the inclusion of an irrelevant negative control (VHH A20.1 or A20.1-Fc, specific for *Clostridioides difficile* toxin A) as an additional test sample. Similarly, serum derived from naïve or pre-immunized animals may also be used as non-inhibitory biological sample controls. In both cases, the absence of neutralization should generate a response comparable to the maximal binding of the bio-spike to ACE2 (Figure 4A,B, see A20.1). On the contrary, Abs with neutralizing capacity will generate a reduction in the signal. If dose–response is evaluated, the user may determine an *IC*_50_ for the test sample (Figure 4A). For improved robustness, we recommend the inclusion of an internal control (e.g., soluble ACE2 or known SARS-CoV-2 nAbs could be used as benchmark) to calibrate and account for experimental variability. Implementation and consistent use of widely available control samples, such as commercially available nAbs, WHO standards and soluble ACE2, would enable users to determine inter-assay and intra-assay coefficients of variation and inter-laboratory variability [36,37].

After the assay is completed and the plates are read by flow cytometry, the data is analyzed using the FlowJo™ software platform (BD Life Sciences). Within the software, cells are gated as described in Figure 3A,B, and the GMFI of PE is extracted for each well. The data is exported to an Excel spreadsheet where the values are used to apply to formula described in 3.3.31 (see Supplementary Data). This formula relates the observed GMFI to the maximal or background control values, enabling determination of relative neutralization percentage.

Depending on the type of the assay, the data is used to plot the single concentrations or to obtain a dose-dependent reduction in the signal that is used to calculate the *IC*_50_ (Figure 4B). This parameter will allow the user to compare Abs in terms of their neutralization potency.

The methodology was further adapted to evaluate the neutralization capacity of monoclonal antibodies and mouse serum against SARS-CoV-2 VOCs. The mutations present in the VOCs increased binding affinity between the human ACE2 receptor and RBDs [38,39], although we obtained comparable results regarding the binding between the bio-spike and ACE2 when using 250 ng/well for each variant tested. A more comprehensive analysis of this assay is shown in Figure 4 of Rossotti et al. (2021) [12], where a collection of 35 VHH-Fcs were assessed against parental Wuhan and several VOCs, where the platform proved useful for illustrating how escape mutations affected the neutralization capacity of the tested antibodies and serums.

## 5. Discussion

The outbreak of COVID-19 incentivized researchers to develop or adapt a plethora of diagnostic methods and research tools that facilitated the evaluation of monoclonal (i.e., mAbs, Ab fragments or VHHs from recombinant sources) and polyclonal samples (i.e., Abs from serum/plasma) for their neutralization activity. Our contribution to this field has been the development of cell-based spike–ACE2 binding assay, employing flow cytometry to expedite the assessment of potential neutralization activity within biological samples that can be performed entirely at CL1. The method proved to be robust and allowed us to measure receptor-blocking Abs found in animals vaccinated against SARS-CoV-2 spike, in addition to screening and quantifying the *IC*_50_ of samples, such as purified mAbs and Ab fragments. Importantly, those results aligned closely with established reference methods, such as pseudotyped virus and authentic live virus neutralization assays. These correlations can be found in our previous works, specifically Supplementary Figure 3 within Akache et al. (2022) [34] and Table 2 and Figure 4A of Rossotti et al. (2021) [12].

The novelty of this assay comes from its core components. The use of cells expressing ACE2 provides maximal realism for receptor context, as opposed to adsorbed or plate-bound ACE2 in methods such as ELISA or SPR. Similarly, the use of a trimeric bio-spike, particularly one with proline stabilization and furin cleavage site ablation, provides a conformationally similar substrate to the pre-fusion spike on the surface of a viral particle [30,31]. As described, the procedure facilitates an assessment of SARS-CoV-2 spike neutralization without handling infectious virus or pseudovirus, as is a requirement for PRNT or pseudotyped lentiviral assays. The utility and versatility of this platform was exemplified by its rapid prediction of authentic neutralization capabilities and its simple adaptation to evaluate the neutralization of a collection of nanobodies [12] and sera [24,25,32,33,34] against viruses other than SARS-CoV-2. These adaptations include SARS-CoV-2 VOCs, SARS-CoV and other potentially zoonotic coronaviruses which also interact with ACE2 as their entry receptor. This was possible by simply using the corresponding biotinylated spike protein generated following the same procedure described in 3.2. Similar adaptations are possible for emerging viral pathogens beyond sarbecoviruses, especially where viral receptor or cell permissiveness has already been determined [40,41]. Assuming that the viral spike sequence is known, this could certainly enhance epidemic or pandemic research responses.

Like other neutralization assays (e.g., pseudotyped lentiviral), our flow cytometry-based approach is ultimately intended to be used as a complementary tool for the screening of nAbs to accommodate throughput requirements or lack of CL2/3 infrastructure. This is ideally suited in a preclinical setting or as a preliminary screening tool used in conjunction with established virus-based assays. Limitations of this technique include the inability to identify nAbs that mediate function after the receptor binding step. Upon receptor binding, the spike must facilitate entry through the endocytosis pathway, requiring conformational changes leading to membrane fusion [29,42,43]. An example of this was the failure to classify a VHH targeting the S2 domain (S2A3) as a neutralizer, which was later identified as a neutralizer in authentic viral neutralization assay and conferred protection within hamster challenge against SARS-CoV-2 [12]. It is worth noting that S2A3 VHH also tested negative in a pseudotyped lentiviral neutralization assay, suggesting possible differences in spike conformation between surrogate systems and the authentic virus. In summary, blocking spike–ACE2 interactions within this assay is indeed a screening tool indicative of neutralization potential but may not identify all possible nAbs, particularly those that achieve neutralization after ACE2 engagement, block conformational changes or membrane fusion events. We strongly recommend confirmation by PRNT and challenge studies as an essential step before therapeutic translation.

Our approach, which uses a random biotinylation method, still has room for improvement. For example, random biotinylation has the potential to invoke steric effects that would impact the conformation and/or binding activity of bio-spike with ACE2. To avoid this, we envision using a site-specific biotinylation approach. The expression of spike protein fused to the AviTAG, which allows enzymatic and site specific biotinylation using *E. coli* biotin ligase BirA, would pave the way for achieving this. An alternative that we have explored is the use of fluorescent labeled trimerization-domain-specific secondary Abs (MRed09, a VHH anti-human resistin domain present in the S used in this study [12]), as opposed to streptavidin-PE. Moreover, the time of the assay could be shortened using a spike that is directly chemically conjugated to a fluorochrome, such as Alexa Fluor^®^ dyes, or fused to a fluorescent protein, such as green fluorescent protein. Though care should still be taken to avoid conformational impacts that may influence the reliability of this assay.

Now, over 5 years since the beginning of the COVID-19 outbreak, the public health concern for SARS-CoV-2 has diminished. Nonetheless, iterations of this methodology will still be useful in the future as fast-screening tools during campaigns to discover neutralizing Abs against SARS-CoV-2-related VOCs or other emergent viral agents.

## Data Availability

Data are available on request.

## Supplementary Materials

The following supporting information can be downloaded at: https://www.mdpi.com/article/10.3390/mps8050104/s1, Figure S1: correspond to the raw data used to generate Figure 4A,B.

## References

[1] Tan C.W., Chia W.N., Qin X., Liu P., Chen M.I., Tiu C., Hu Z., Chen V.C., Young B.E., Sia W.R. (2020). A SARS-CoV-2 surrogate virus neutralization test based on antibody-mediated blockage of ACE2-spike protein-protein interaction. Nat. Biotechnol..

[2] Maghsood F., Dashti N., Bahadori T., Golsaz-Shirazi F., Moog C., Amiri M.M., Shokri F. (2025). Comparative assessment of four virus neutralization assays for detection of SARS-CoV-2 neutralizing antibodies. Anal. Biochem..

[3] Sholukh A.M., Fiore-Gartland A., Ford E.S., Miner M.D., Hou Y.J., Tse L.V., Kaiser H., Zhu H., Lu J., Madarampalli B. (2021). Evaluation of Cell-Based and Surrogate SARS-CoV-2 Neutralization Assays. J. Clin. Microbiol..

[4] Ali D.W., Bartlett M.L., Heger C.D., Ramirez F., Johnson L., Schully K.L., Laing E.D., Wang W., Weiss C.D., Goguet E. (2024). Automated and virus variant-programmable surrogate test qualitatively compares to the gold standard SARS-CoV-2 neutralization assay. npj Viruses.

[5] Streif S., Baeumner A.J. (2025). Advances in Surrogate Neutralization Tests for High-Throughput Screening and the Point-of-Care. Anal. Chem..

[6] Nie J., Li Q., Wu J., Zhao C., Hao H., Liu H., Zhang L., Nie L., Qin H., Wang M. (2020). Establishment and validation of a pseudovirus neutralization assay for SARS-CoV-2. Emerg. Microbes Infect..

[7] Lim J.Y., Fiore A., Le B., Minzer C., White H., Burinski K., Janwari H., Wright D., Perebikovsky S., Davis R. (2025). Development and Validation of Novel Cell-free Direct Neutralization Assay for SARS-CoV-2. J. Virol. Methods.

[8] Marien J., Michiels J., Heyndrickx L., Nkuba-Ndaye A., Ceulemans A., Bartholomeeusen K., Madinga J., Mbala-Kingebeni P., Vanlerberghe V., Ahuka-Mundeke S. (2021). Evaluation of a surrogate virus neutralization test for high-throughput serosurveillance of SARS-CoV-2. J. Virol. Methods.

[9] Nie J., Li Q., Wu J., Zhao C., Hao H., Liu H., Zhang L., Nie L., Qin H., Wang M. (2020). Quantification of SARS-CoV-2 neutralizing antibody by a pseudotyped virus-based assay. Nat. Protoc..

[10] Walker S.N., Chokkalingam N., Reuschel E.L., Purwar M., Xu Z., Gary E.N., Kim K.Y., Helble M., Schultheis K., Walters J. (2020). SARS-CoV-2 Assays To Detect Functional Antibody Responses That Block ACE2 Recognition in Vaccinated Animals and Infected Patients. J. Clin. Microbiol..

[11] Abe K.T., Li Z., Samson R., Samavarchi-Tehrani P., Valcourt E.J., Wood H., Budylowski P., Dupuis A.P., Girardin R.C., Rathod B. (2020). A simple protein-based surrogate neutralization assay for SARS-CoV-2. JCI Insight.

[12] Rossotti M.A., van Faassen H., Tran A.T., Sheff J., Sandhu J.K., Duque D., Hewitt M., Wen X., Bavananthasivam J., Beitari S. (2022). Arsenal of nanobodies shows broad-spectrum neutralization against SARS-CoV-2 variants of concern in vitro and in vivo in hamster models. Commun. Biol..

[13] Colwill K., Galipeau Y., Stuible M., Gervais C., Arnold C., Rathod B., Abe K.T., Wang J.H., Pasculescu A., Maltseva M. (2022). A scalable serology solution for profiling humoral immune responses to SARS-CoV-2 infection and vaccination. Clin. Transl. Immunol..

[14] Egia-Mendikute L., Bosch A., Prieto-Fernandez E., Vila-Vecilla L., Zanetti S.R., Lee S.Y., Jimenez-Lasheras B., Garcia Del Rio A., Antonana-Vildosola A., de Blas A. (2022). A flow cytometry-based neutralization assay for simultaneous evaluation of blocking antibodies against SARS-CoV-2 variants. Front. Immunol..

[15] Bloch E., Garcia L., Donnadieu F., Rosado J., Planas D., Bruel T., Hocqueloux L., Prazuck T., Schwartz O., Tondeur L. (2025). Multiplex ACE2-RBD binding inhibition assay: An integrated tool for assessing neutralizing antibodies to SARS-CoV-2 variants and protection against breakthrough infections. J. Immunol. Methods.

[16] Yao W., Li Y., Sun H., Ma D., Tang X., Zeng A., Huang F. (2025). Characterization of key spike RBD residues influencing SARS-CoV-2 variant adaptation to avian ACE2. Front. Cell. Infect. Microbiol..

[17] Izac J.R., Kwee E.J., Tian L., Elsheikh E., Gaigalas A.K., Elliott J.T., Wang L. (2023). Development of a Cell-Based SARS-CoV-2 Pseudovirus Neutralization Assay Using Imaging and Flow Cytometry Analysis. Int. J. Mol. Sci..

[18] Cao Y., Sun Y., Tian X., Bai Z., Gong Y., Qi J., Liu D., Liu W., Li J. (2020). Analysis of ACE2 Gene-Encoded Proteins Across Mammalian Species. Front. Vet. Sci..

[19] Melin A.D., Janiak M.C., Marrone F., Arora P.S., Higham J.P. (2020). Comparative ACE2 variation and primate COVID-19 risk. Commun. Biol..

[20] Ma C., Gong C. (2021). ACE2 models of frequently contacted animals provide clues of their SARS-CoV-2 S protein affinity and viral susceptibility. J. Med. Virol..

[21] Pi-Estopinan F., Perez M.T., Fraga A., Bergado G., Diaz G.D., Orosa I., Diaz M., Solozabal J.A., Rodriguez L.M., Garcia-Rivera D. (2022). A cell-based ELISA as surrogate of virus neutralization assay for RBD SARS-CoV-2 specific antibodies. Vaccine.

[22] Yang J., Wang W., Chen Z., Lu S., Yang F., Bi Z., Bao L., Mo F., Li X., Huang Y. (2020). A vaccine targeting the RBD of the S protein of SARS-CoV-2 induces protective immunity. Nature.

[23] Sherman E.J., Emmer B.T. (2021). ACE2 protein expression within isogenic cell lines is heterogeneous and associated with distinct transcriptomes. Sci. Rep..

[24] Renner T.M., Akache B., Stuible M., Rohani N., Cepero-Donates Y., Deschatelets L., Dudani R., Harrison B.A., Baardsnes J., Koyuturk I. (2023). Tuning the immune response: Sulfated archaeal glycolipid archaeosomes as an effective vaccine adjuvant for induction of humoral and cell-mediated immunity towards the SARS-CoV-2 Omicron variant of concern. Front. Immunol..

[25] Renner T.M., Stuible M., Rossotti M.A., Rohani N., Cepero-Donates Y., Sauvageau J., Deschatelets L., Dudani R., Harrison B.A., Baardsnes J. (2025). Modifying the glycosylation profile of SARS-CoV-2 spike-based subunit vaccines alters focusing of the humoral immune response in a mouse model. Commun. Med..

[26] Arias-Arias J.L., Monturiol-Gross L., Corrales-Aguilar E. (2025). A Live-Cell Imaging-Based Fluorescent SARS-CoV-2 Neutralization Assay by Antibody-Mediated Blockage of Receptor Binding Domain-ACE2 Interaction. Biotech.

[27] Bermudez-Abreut E., Fundora-Barrios T., Hernandez Fernandez D.R., Noa Romero E., Fraga-Quintero A., Casadesus Pazos A.V., Fernandez-Marrero B., Plasencia Iglesias C.A., Clavel Perez M., Sosa Aguiar K. (2025). Antiviral activity of an ACE2-Fc fusion protein against SARS-CoV-2 and its variants. PLoS ONE.

[28] Dashti N., Golsaz-Shirazi F., Soltanghoraee H., Zarnani A.H., Mohammadi M., Imani D., Jeddi-Tehrani M., Amiri M.M., Shokri F. (2024). Preclinical assessment of a recombinant RBD-Fc fusion protein as SARS-CoV-2 candidate vaccine. Eur. J. Microbiol. Immunol..

[29] Chen Y., Zhao X., Zhou H., Zhu H., Jiang S., Wang P. (2023). Broadly neutralizing antibodies to SARS-CoV-2 and other human coronaviruses. Nat. Rev. Immunol..

[30] Amanat F., Strohmeier S., Rathnasinghe R., Schotsaert M., Coughlan L., Garcia-Sastre A., Krammer F. (2021). Introduction of Two Prolines and Removal of the Polybasic Cleavage Site Lead to Higher Efficacy of a Recombinant Spike-Based SARS-CoV-2 Vaccine in the Mouse Model. mBio.

[31] Stuible M., Gervais C., Lord-Dufour S., Perret S., L’Abbe D., Schrag J., St-Laurent G., Durocher Y. (2021). Rapid, high-yield production of full-length SARS-CoV-2 spike ectodomain by transient gene expression in CHO cells. J. Biotechnol..

[32] Akache B., Renner T.M., Tran A., Deschatelets L., Dudani R., Harrison B.A., Duque D., Haukenfrers J., Rossotti M.A., Gaudreault F. (2021). Immunogenic and efficacious SARS-CoV-2 vaccine based on resistin-trimerized spike antigen SmT1 and SLA archaeosome adjuvant. Sci. Rep..

[33] Renner T.M., Stuible M., Cass B., Perret S., Guimond J., Lord-Dufour S., McCluskie M.J., Durocher Y., Akache B. (2024). Reduced cross-protective potential of Omicron compared to ancestral SARS-CoV-2 spike vaccines against potentially zoonotic coronaviruses. npj Viruses.

[34] Akache B., Renner T.M., Stuible M., Rohani N., Cepero-Donates Y., Deschatelets L., Dudani R., Harrison B.A., Gervais C., Hill J.J. (2022). Immunogenicity of SARS-CoV-2 spike antigens derived from Beta & Delta variants of concern. npj Vaccines.

[35] Stocks B., Thibeault M.-P., Bates J., Lord-Dufour S., Gervais C., Stuible M., Durocher Y., Melanson J. (2021). SMT1-1: SARS-CoV-2 Spike Glycoprotein Reference Material SMT1-1.

[36] Kricka L.J., Master S.R. (2008). Validation and quality control of protein microarray-based analytical methods. Mol. Biotechnol..

[37] Kim H., Shin S., Hwang S.H., Kim M., Cho Y.U., Jang S. (2023). Validation of High-sensitivity Flow Cytometry for Reliable Immune Cell Analysis in Real-world Laboratory Settings. Ann. Lab. Med..

[38] Tachibana K., Nakamura Y., Do T.L., Kihara T., Kawada H., Yamamoto N., Ando K. (2024). Mutations in the SARS-CoV-2 spike proteins affected the ACE2-binding affinity during the development of Omicron pandemic variants. Biochem. Biophys. Res. Commun..

[39] Sultana N., Nagesha S.N., Reddy C.N.L., Ramesh B.N., Shyamalamma S., Shashidhara K.S., Satish K.M., Pradeep C., Vidyadhar G.D. (2024). Computational analysis of affinity dynamics between the variants of SARS-CoV-2 spike protein (RBD) and human ACE-2 receptor. Virol. J..

[40] Barrass S.V., Butcher S.J. (2020). Advances in high-throughput methods for the identification of virus receptors. Med. Microbiol. Immunol..

[41] Valero-Rello A., Baeza-Delgado C., Andreu-Moreno I., Sanjuan R. (2024). Cellular receptors for mammalian viruses. PLoS Pathog..

[42] De Cae S., Van Molle I., van Schie L., Shoemaker S.R., Deckers J., Debeuf N., Lameire S., Nerinckx W., Roose K., Fijalkowska D. (2025). Ultrapotent SARS coronavirus-neutralizing single-domain antibodies that clamp the spike at its base. Nat. Commun..

[43] Lee W.S., Taiaroa G., Esterbauer R., Conlan M., Neil J., Gartner M., Smith M., Wang M., Shepherd R., Tran T. (2025). Potent neutralising monoclonal antibodies targeting the spike of NL63 coronavirus. npj Viruses.

